# Phase Transformation after Heat Treatment of Cr-Ni Stainless Steel Powder for 3D Printing

**DOI:** 10.3390/ma15155343

**Published:** 2022-08-03

**Authors:** Karla Čech Barabaszová, Aleš Slíva, Gabriela Kratošová, Sylva Holešová, Anastasia Volodarskaja, Tugrul Cetinkaya, Silvie Brožová, Libor Kozubek, Gražyna Simha Martynková

**Affiliations:** 1 Nanotechnology Centre, CEET, VŠB—Technical University of Ostrava, 17. Listopadu 15/2172, 708 00 Ostrava, Czech Republic; karla.cech.barabaszova@vsb.cz (K.Č.B.); gabriela.kratosova@vsb.cz (G.K.); sylva.holesova@vsb.cz (S.H.); 2 Institute of Transport, Faculty of Mechanical Engineering, VŠB—Technical University of Ostrava, 17. Listopadu 15/2172, 708 00 Ostrava, Czech Republic; ales.sliva@vsb.cz; 3 Department of Material Engineering, Faculty of Materials Science and Technology, VŠB—Technical University of Ostrava, 17. Listopadu 15/2172, 708 00 Ostrava, Czech Republic; anastasia.volodarskaja@vsb.cz; 4 Department of Metallurgical and Materials, Engineering Faculty, Esentepe Kampüsü, Sakarya University, Kemalpaşa, Üniversite Cd., 54050 Serdivan, Turkey; tcetinkaya@sakarya.edu.tr; 5 Department of Non-Ferrous Metal, Refining and Recycling, Faculty of Materials Science and Technology, VŠB—Technical University of Ostrava, 17. Listopadu 15/2172, 708 00 Ostrava, Czech Republic; silvie.brozova@vsb.cz; 6 Brebeck Composite s.r.o. 1718, Volenská, 739 34 Šenov, Czech Republic; kozubek@brebeckcomposite.com

**Keywords:** 316 L stainless steel powder, 3D printing, morphology, particle size, heat treatment, oxidation

## Abstract

Today, Ni-Cr steel is used for advanced applications in the high-temperature and electrical industries, medical equipment, food industry, agriculture and is applied in food and beverage packaging and kitchenware, automotive or mesh. A study of input steel powder from various stages of the recycling process intended for 3D printing was conducted. In addition to the precise evaluation of the morphology, particle size and composition of the powders used for laser 3D printing, special testing and evaluation of the heat-treated powders were carried out. Heat treatment up to 950 °C in an air atmosphere revealed the properties of powders that can appear during laser sintering. The powders in the oxidizing atmosphere change the phase composition and the original FeNiCr stainless steel changes to a two-phase system of Fe_3_Ni and Cr_2_O_3_, as evaluated by X-ray diffraction analysis. Observation of the morphology showed the separation of the oxidic phase in the sense of a brittle shell. The inner part of the powder particle is a porous compact core. The particle size is generally reduced due to the peeling of the oxide shell. This effect can be critical to 3D printing processing, causing defects on the printed parts, as well as reducing the usability of the precursor powder and can also change the properties of the printed part.

## 1. Introduction

Metal additive manufacturing (AM) is growing in importance in many areas of life and simultaneously, the need for research is rising. Significant areas within this field are aerospace, automotive, military [1,2], medical and dental areas [3].

Along with the production processes, effective utilization and recycling must be taken into account to ensure economic reliability [4,5,6,7].

Metal AM precursor materials present the most important part in the manufacturing process, and they should be watched for changes in their chemical and phase composition, as well as morphology and particle size of the powder [8]. The changes can happen due to mechanical or thermal effects [9]. The thermal history, particularly the solidification rate, temperature gradient, and consequently the cooling rate, associated with any AM part deeply influences the formation of volumetric defects, and therefore final microstructure. For example, a more homogenized microstructure with fewer volumetric defects is produced when the part is large or the interlayer time interval is short, or when the process parameters (such as laser power, scan speed and scan strategy) are optimized. Altering the thermal history also extends to the pre-heating of the build platform, which has been found to be effective in varying the microstructures of both electron beam powder bed fused (EB-PBF) Inconel 718 or Ti_6_Al_4_V and laser beam powder bed fused (LB-PBF) Al-Mg(-Sc)-Zr samples. The static strength of AM materials is typically higher than the wrought counterparts due to the finer microstructure, resulting from the high solidification rate [10,11,12].

Chromium-nickel austenitic stainless steels of the 300 series are one of the most widely used materials in the world due to their good ductility, weldability, and high corrosion resistance. These features make it a great candidate for implementation in several industries, such as the medical field for surgical assistance, endoscopic surgery, or orthopedics; in the aerospace industry for producing mechanical parts; in the automobile industry for corrosion-resistant parts [13] but also for making watches and jewelry [14].

Stainless steel printing is very accurate because of the fine coating resolution (30–40 µm) and the laser’s accuracy [15,16]. Unlike polymer powder sintering, stainless steel printing requires adding base structures to attach the part to the board and to strengthen distinctive geometry-like overhangs [17]. The bases themselves are made from the same powder as the piece and will be taken off afterwards. Smooth and shiny surfaces can be acquired after printing via the finishing steps. Pieces can be machined, drilled, welded, electro-eroded, granulated, polished, and coated [18]. Compared to the other metal 3D printing materials, stainless steel is the smoothest material.

One of the basic issues during production is powder recycling. Degradation of powders, resulting from repeated reuses, was found to be a widespread problem; components produced from heavily reused powders are typically of a lower quality, eventually rendering the powder unusable in additive manufacturing. Powder degradation was found to be dependent on many variables, preventing the identification of a definitive end-of-life point for powders. The most accurate method of determining powder quality was found to be the production and analysis of components using these powders [19,20].

The aim of the study was to evaluate the powders used for 3D printing in their original state from production and after heat treatment to observe the processing conditions. As oxidation of the surface of spherical particles of the powders can be significant, the evaluation of new phases is important for their practical usage. Heat treatment up to 950 °C was performed for all the studied powders, with the aim of observing the most sensitive region of the laser-treated material. Observation of the shapes, distribution of particle size, changes in chemical composition and the general degradation of the powder was carried out using several analytical techniques. The suggestion for the additive technology is given.

## 2. Materials and Additive Manufacturing Conditions

For the study, the metallic powder used for 3D printing was collected directly from the production line of a collaborating company. Three powder samples from various stages of production were studied; one sample was fresh (later FRESH) powder that had not been used for printing yet, then powder used 3 times (later 3×) and finally, powder used 6 times (later 6×) that was not used again for printing later.

These powders were screened on a sieve with a mesh size of 53 mm for utilization and the original powder was used in a grain size range of 15–45 mm.

To explain the origin of the powders, a short description of the 3D printing process is given; however, the printed parts are not presented in this study. The process for the 3D printing of metallic powder in our case is as follows. The printer used for these tests is the EOS M280 with a laser power of 400 W, Yb-fiber laser, scan speed 7 m/s and focus diameter 0.1–0.5 mm. The printer is filled with new powder and printing is started. The sample of new powder is poured into a container and after printing, the leftovers of the remaining powder (overflow and powder from around the printed samples) is sieved on the above-described 53 mm sieve and undersized powder is returned to the printer. This continues until the powder is used 3 times (sample 3×) and used 6 times (sample 6×).

A new batch is added during the second print, which means that the whole batch is regenerated. The normal print job is 60–100 h. This piece has ~10 kg and the printer can hold ~100–110 kg of powder (so after 2–3 pieces, one would be missing 1/3 of the powder and one will not complete a job); therefore, the printer is usually filled to “full” to print without interruption, if it is possible. The size of the working space of the printer does not correspond to the size of the dust container, that is, it is about 50% smaller.

The simulation of the extreme conditions using heat treatment at 950 °C was performed along with the set of relevant analyses. The temperature was selected based on the observed temperature changes during the thermal analysis, which was found in range 900–1000 °C (presented later in the Section 3.4). The heat treatment of all samples was performed in a muffle furnace in air atmosphere, where samples were placed at the beginning of the process and the heating process started, with a rate of 5 °C/min from room temperature up to 950 °C, remaining at this temperature for 30 min, followed by controlled cooling of 5 °C/min until 200 °C and after the natural cooling mode. The sample was removed from the furnace and kept in a tight glass container.

### Characterization Methods

The selected characterization techniques were employed to obtain a picture for the studied metallic powders.

One of the basic methods for the powder study was the estimation of powder particle size distribution. The particle size of the powder samples was determined using the HORIBA laser diffraction particle size analyzer (LA-950 instrument, Kyoto, Japan) with two short-wavelength blue and red-light sources, in conjunction with forward and backscatter detection, to enhance sizing performance in the range 0.01–3000 µm. The particle size analyses were conducted with the refractive indices 2.900 (for iron oxide), and 1.33 (for water). Each sample was measured three times. Selected samples were analyzed for particle size and shape using the HORIBA PSA300 image analyzer.

The morphology of the powder samples was investigated using the scanning electron microscope (SEM) JEOL JSM-7610F Plus, Tokyo, Japan with a Schottky cathode. Powder samples were prepared on stubs with carbon tape and were directly observed without coating in a high vacuum chamber. Elemental composition analysis and mapping were performed using the energy-dispersive X-ray spectroscope (EDS) AZtec Ultima Max 65 (Oxford Instruments, Abingdon, UK). The same analyzer and software were used for particles size distribution evaluation after the contrast/brightness and threshold setting (AZtec feature particle analysis—AFPA).

The X-ray powder diffraction (XRD) analysis of the powder samples was performed using the diffractometer RIGAKU Ultima IV, Tokyo, Japan (CuKα radiation, Ni-Kβ filter, Bragg-Brentano arrangement, scintillation detector). Samples in the standard holder were measured in ambient atmosphere, with the operating conditions 40 kV and 40 mA. Samples were measured in the range 2θ 1.5–70°, with a scanning rate of 2.7°/min and step width of 0.02°. Phase analysis was evaluated by database PDF-2 (release 2011). Figures of the XRD patterns were drawn using Origin8Pro software.

The thermal analysis of the nontreated powder samples was performed using a Setsys 24 Evolution Setaram thermal analyzer (Setaram, France). The thermal curves were recorded under the following conditions: Ar atmosphere (75 mL/min), final temperature 1200 °C, heating rate of 5 °C/min and sample mass about 14 mg.

## 3. Results and Discussion

The following chapters will focus on characterization of all the studied samples. It is very important to perform detailed evaluation of the particle size of the production powders and observe their shapes and morphology, since these are the most important parameters for optimal sintration during the 3D printing of the product. Other factors for efficient printing include the stable chemical composition of the powder, meaning the elimination of any reaction with oxygen or moisture.

Table 1 refers to the chemical composition of the stainless-steel powder used in the study, as given in the production list.

### 3.1. Morphology and Chemical Composition of Stainless-Steel Powders

**Figure 1:**
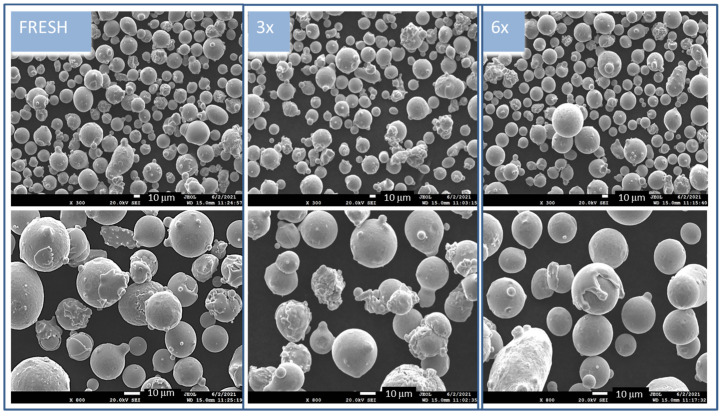
Morphology of all nontreated powders in two magnifications—at 300× in upper row images, and in more detail, focusing on individual particles, at 800× (bottom row images) in the mode of secondary electrons (SE); scale bar indicated in white color is 10 mm.

Electron micrographs of the original sample FRESH and both the used 3× and 6× samples are presented in Figure 1. The samples before heat treatment were imagined at two magnifications, where 300× magnification presents the size distribution of the powder particles and 800× magnification shows the character of the particles. The particles are characterized by smooth and compact surfaces; however, certain particles are decorated with small satellite particles. The FRESH and 3× used samples have many agglomerates that are especially visible at 800× magnification. The sample of 6× used powder is exposes a melted “slapped cap” on the round particles. This phenomenon is rarely visible for particles in the powder used 3×.

**Figure 2:**
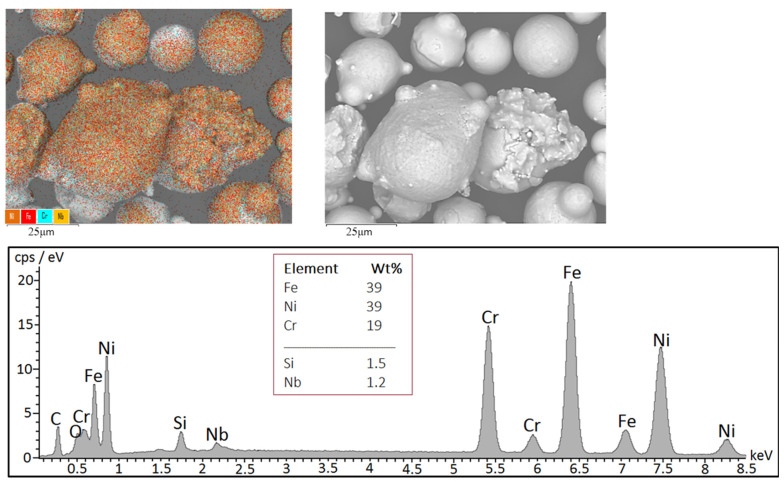
Elemental mapping of particle surfaces (**left**) in FRESH sample, electron image of particles in BSE mode (**right**) and characteristic X-ray spectrum with the evaluation of main elements.

The morphology observation was followed by the analyses of the chemical composition and the distribution of the elements related to the particle’s surface. Chemical composition was analyzed using EDS. The elements map in Figure 2 shows uniform distribution of all the analyzed elements in the FRESH sample, where Fe and Ni show equal weight quantity and Cr is half of this amount. There are minor elements, such as Si, and Nb (carbon was not included in the quantification due to the known inaccuracy of the EDS method in the quantification of light elements and its presence in the carbon tape). In Figure 2, on the right, we can observe the electron image of the particles displayed in the backscattered electron mode (BSE), where material contrast is not evident on the presented particles, which also indicates an even distribution of the majority elements in the material.

**Figure 3:**
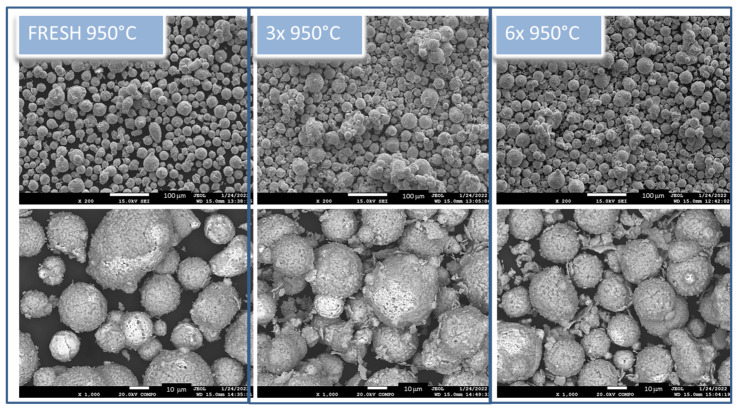
Morphology of all thermally treated powders in two magnifications: 200× (**upper**) scale bar 1000 mm and 1000× (**bottom** images) scale bar 10 mm. In lower magnification, the morphology changes and agglomeration of particles is visible in the mode of secondary electrons. Chemical composition changes and Z-contrast are better observed on magnified pictures in the mode of backscattered electrons.

The same evaluation of morphology and elemental distribution was performed for the heat-treated samples. Thermally treated samples exposed different surface morphology, as shown in Figure 3. Even through a rough surface, we can still observe the small satellite particles. In simple terms, the powder particles are composed as “core-shell” particles that change the material’s appearance. In the magnified images, there are clearly visible small 2D particles, and these are peeled out from the basic-core material of the particle. The core of the particle is smooth and compact, with compact porous morphology, while the shell is also porous but brittle and peels out. The shells peel more in the case of the 6× 950 °C sample, where a high number of broken shells in the sample could be observed. Particles after heat treatment did not change size significantly. However, we can observe aggregated particles with visible borders between particles that seem to be breakable.

**Figure 4:**
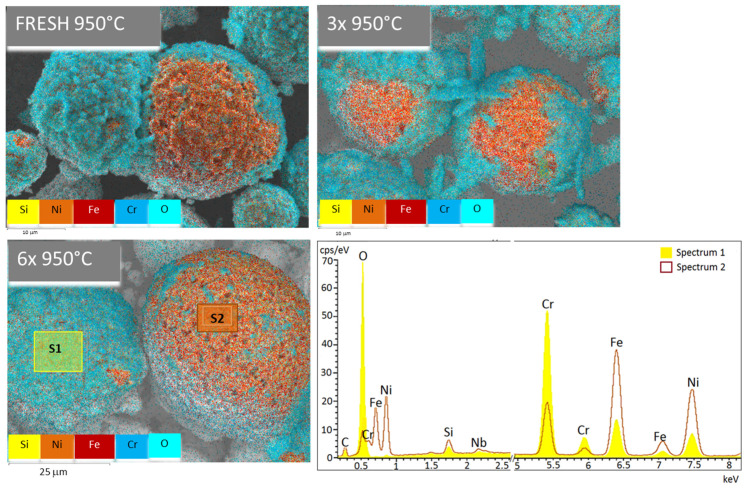
Mapping of particle surfaces along with spectrum evaluation of main elements for sample 6×. From the mapping of the two areas of interest—the shell (S1) and the core (S2)—and from the interpolation of both spectra, a significant effect of the temperature treatment is evident. The basic core material is FeNi alloy, and the shell is made of CrO oxides.

By analyzing the elemental composition of the heat-treated samples, we have found out that the basic core material is FeNi alloy and the shell is made of CrO oxides (Figure 4). The shell parts are more porous. It can be observed that the shell part becomes larger for the samples used 6×, where a spectrum of elements was acquired. The analysis confirms that the shell (yellow spectrum 1) is composed of Cr and O elements, and inner core part (orange spectrum 2) is mainly related to Fe and Ni elements and also O and Cr, but is radically smaller than for the shell part. There are traces of Si and Nb in both parts in similar quantity.

### 3.2. Particle Size Analysis

One of the most important characteristics of the powdered matter is particle size evaluation. The characterization of powder was carried out using the following two techniques: (1) SEM analysis equipped with Aztec Feature Particle Analysis (AFPA) software and (2) the laser diffraction method of whole bulk material.

#### 3.2.1. Electron Microscopy Image Processing via AFPA

**Figure 5:**
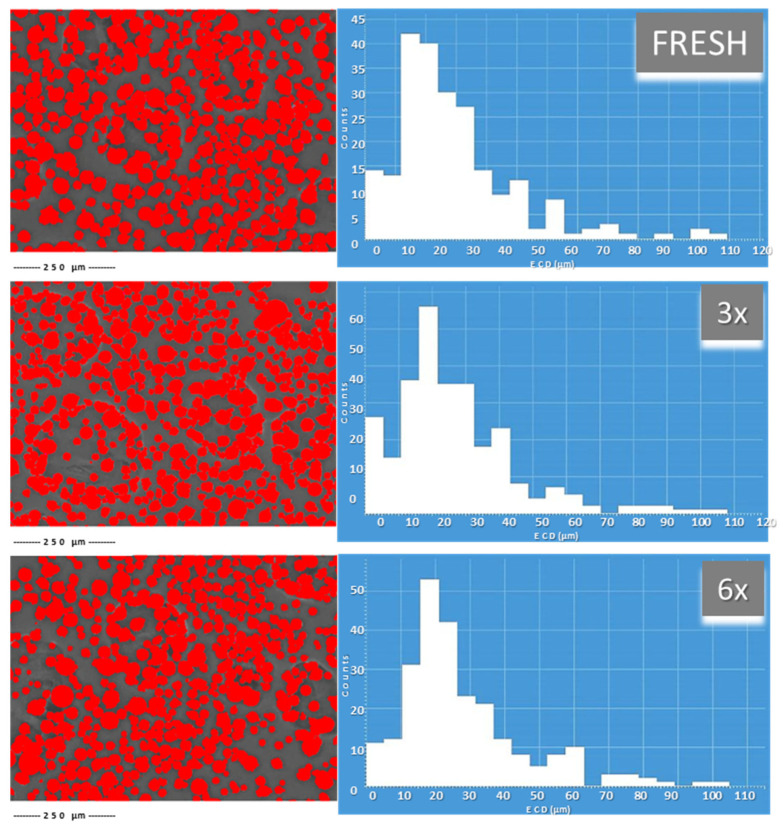
AFPA processing of BSE images for all powders before heat treatment and particle size distribution.

Software-processed electron images from the BSE mode using AFPA showed changing particle sizes in the powder FRESH compared to the 3× or 6× used samples. The histograms of all the samples (Figure 5) showed relatively uniform particle size distribution in the range 10–50 μm, where the majority of the powder particles lay. Powders used 3× and 6× showed a higher frequency of particles with bigger sizes of about 80 μm and above, compared to the FRESH sample.

#### 3.2.2. Particle Size Distribution of Whole Powder

**Figure 6:**
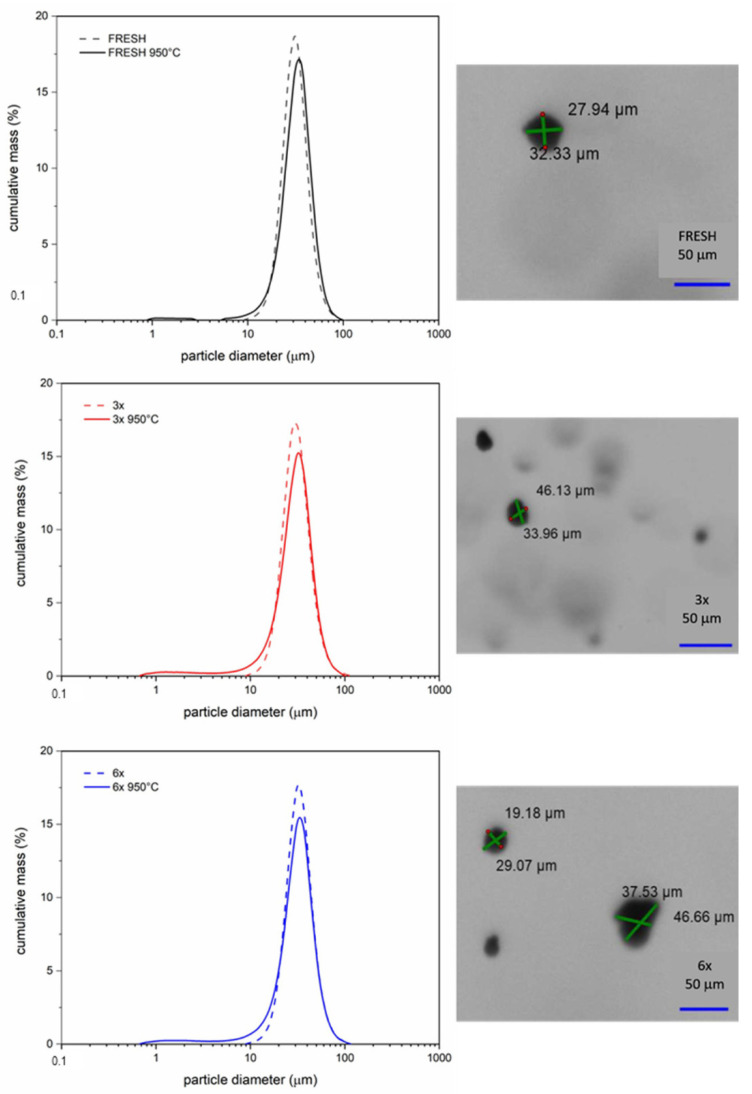
Particle size distributions (lognormal distributions) of the FRESH, 3× and 6× used samples and temperature treatment of the FRESH 950 °C, 3× 950 °C and 6× 950 °C samples. The optical images of FRESH, 3× and 6× samples.

The particle size parameters measured using the laser diffraction method in the liquid medium are shown in Table 2. The parameters that express the particle size distribution data were the volume-weighted mean diameter (De Brouckere mean diameter, d_43_), the maximum diameter value of the peak (dm) and d_10_ and d_90_ represent 10% and 90% of the particles, respectively, in the powders and span value (the width of the size distribution (d_90_ − d_10_)/d_50_). The particle size distributions of all the measured powder samples are shown in Figure 6. The images of the FRESH, 3× and 6× used samples taken during the particle size measurements are also shown, which demonstrate the shape and size variability of the analyzed samples.

The FRESH, 3× and 6× used samples showed monomodal particle size distributions with identical span value, with particle sizes in the range of 18.74 to 46.47 μm. From the d_43_ and dm values, it is evident that the 3× used sample shows a lower particle size and vice versa, the 6× used sample shows higher values than the original FRESH sample. The optical images of the FRESH, 3× and 6× used samples, taken during the particle size analyses, show a difference in the particles’ shapes, which are formed by satellites on the original particles’ surfaces (shown in SEM images). Evident satellites are located on the particles’ surfaces, with larger diameters in the sample bulk.

The particle sizes of the heat-treated samples are smaller by about 1.5 μm, which adequately decreased the % frequency of individual fractions in the sample volumes. This reduction is due to the formation of new size fractions in all the heat-treated samples. In the case of the FRESH 950 °C sample, a new fraction was formed in the range of values of 1–2.599 μm, which forms a separate distribution curve. The distribution curves of 3× 950 °C and 6× 950 °C samples are continuous, starting at 0.766 μm, with the finest fractions in the range 0.766–14.75 μm (15.34 μm, respectively), making up less than 1% of the sample volume. These changes in the distribution curves indicate the mutual interaction of very fine fractions with larger fractions. These behavior changes are due to the surface oxidation of the powder samples, when chromium oxides are released from the particle surfaces, as confirmed by EDS (SEM) and XRD analyses.

The results show that the apparent activation energy is significantly affected by particle size in such a way that the apparent activation energy increases along with the particle size. From a physical viewpoint, the contact area among the particles will increase as the particle size decreases for a given volume. The larger contact areas lead to better diffusion among particles. Therefore, the powder sample of a smaller particle size will require lower activation energy for sintering. The results imply that the interactions between a pair of particles are enhanced by decreasing the particle size [21].

### 3.3. Phase Analysis Results

Based on the elemental analysis obtained from EDS, phase analysis was performed. XRD analysis for both the non-treated and treated material exposed the phases of the samples. The non-treated sample was a pure steel material without impurities.

**Figure 7:**
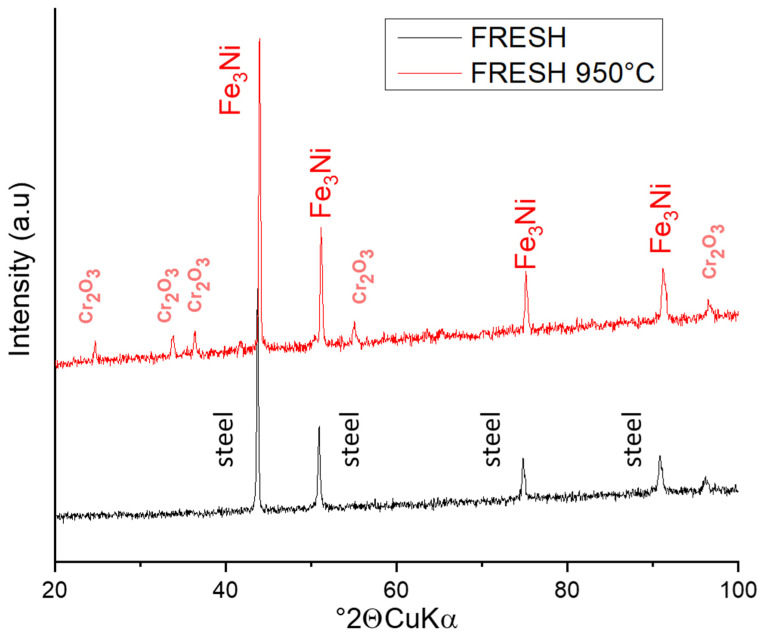
XRD pattern of FRESH sample before and after treatment 950 °C. The most intensive peaks are noted with analyzed phase name.

**Figure 8:**
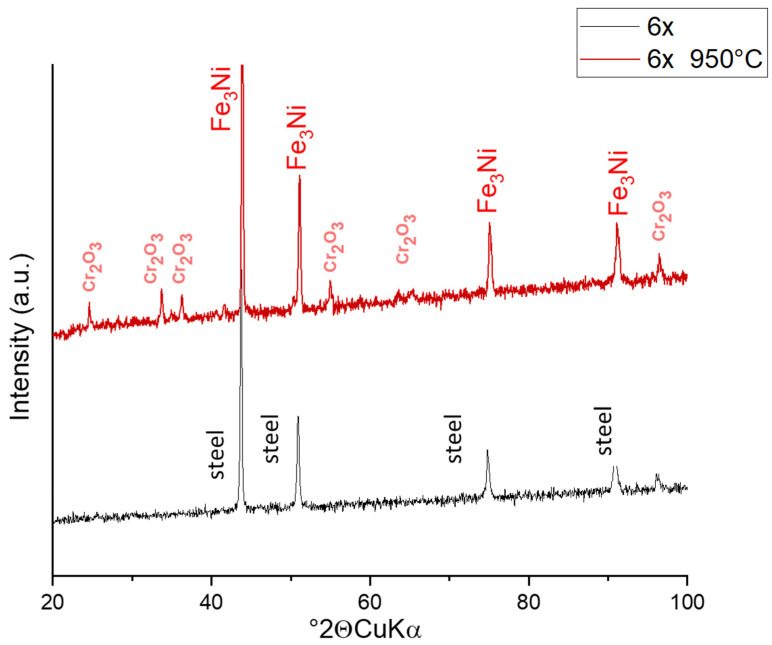
XRD pattern of sample 6× used before and after treatment. The most intensive peaks are noted with analyzed phase name.

After heat treatment, the phases in the material changed radically and one phase system exposed new peaks of a different phase. The changes in the sample FRESH (Figure 7) and 3× are comparable and in sample 6×, the intensities of the evaluated phases differ slightly; therefore, these patterns are shown (Figure 8).

The phase containing Fe and Ni was evaluated as Fe_3_Ni teanite, JCPDS PDF card 01-071-8325, cubic system, Fm-3m (225), a = 3.575Å. The phase with Cr is Cr_2_O_3_ eskolaite, JCPDS PDF 01-084-1616, trigonal system, R-3c (167), a, c = 4.952 Å, c = 13.598 Å, which are the parameters of the cell. The character of the peaks corresponds to the evidently lower crystalline appearance of Cr-oxide and the better ordered structure of the teanite.

The eskolaite [19] in the sample 6× shows higher intensity of the phase peaks, which correlates with the results from SEM observation (Figure 3), where higher exfoliation of oxides was observed. Cr_2_O_3_ is the only solid chromium oxide phase that is stable at temperatures above 500 °C. At temperatures below 500 °C, several other oxygen-rich phases can exist in the composition range Cr_2_O_3_–CrO_3_. Cr_2_O_3_ is an intrinsic semiconductor, whose conductance is independent of oxygen partial pressure at high temperatures (>1000 °C), whereas at lower temperatures, the oxide is an extrinsic p-type semiconductor [22]. The original FeNiCr is, in a highly dense state, electrically conductive [19].

### 3.4. Thermal Analysis of Non-Treated Samples

**Figure 9:**
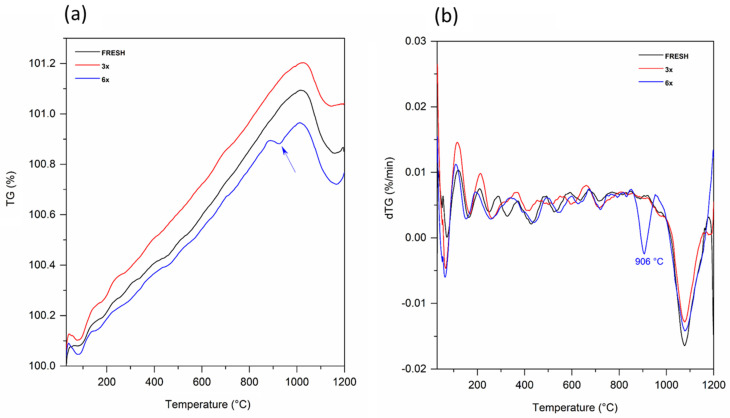
Thermal analysis of nontreated samples: (**a**) TG and (**b**) dTG curves. The significant temperature changes are marked.

Thermogravimetry was performed for the nontreated samples. TG curves in Figure 9a showing the increase in the weight across the temperature range, up to approx. 1020 °C. Then weight drops for all the samples. This behavior on the dTG curves (Figure 9b) belongs to the peaks at 1077 °C for FRESH and 3× and 1079 °C for 6×, which probably indicate the partial phase transition to an alloy and oxide separation in the powdered material.

The thermal curves show the special behavior of the sample 6× used powder, because we can observe a unique change at 905 °C. We can assume that this change can be attributed to the creation of a greater amount of Cr_2_O_3_ in this sample (after multiple usage).

## 4. Conclusions

The application-inspired study of Cr-Ni stainless steel with a broad application range, including the high-temperature and electric industry, medical devices, construction, chemistry, food industry, agriculture, and aeronautics, is presented. The powder used for 3D printing was studied for the morphology, particle size and structure changes that develop during the regular production cycle. An additional observation after heat treatment was performed.

Particles that show morphological changes after being used 6×, particularly a slapped cape of partially melted matter, are visible on the original spherical particles.

The small size fraction from the original sample is reduced during utilization in the printing process; therefore, for the sample used 6×, the small fraction is at the minimum value. This presumption was confirmed by evaluating the bulk particle size where the 6× used sample was shown to be on average larger than the starting powder. The elimination of small particles is the reason for lower sintration properties.

Thermal analysis of the powders shows evident changes at about 1070 °C, which is the same for all the analyzed samples, and must be related to phase transition. The sample 6× has special thermal behavior at 920 °C, which is related to the material changes caused by laser processing of the residual powder that gradually appears. This result is related to the changes observed in the samples after laboratory heat treatment.

After laboratory heat treatment of all the studied samples, changes in morphology, phase composition and particle size distribution are visible.

The heat treatment in an oxidizing atmosphere shows that the powder changes dramatically, and powder particles become core-shell-like. The creation of a special oxidic brittle shell made of Cr_2_O_3_ was visualized via SEM analysis and the inner part, which appeared as a porous sponge, was detected as the FeNi alloy. The particles size slightly decreased on average by 1.5 mm and the span of particle size distribution was wider.

This assessment is important for manufacturing where the technology runs under test conditions, including oxygen atmospheres and local temperatures above 900°C, and even negligible oxidation could affect the conductivity and product properties.

## Figures and Tables

**Table 1:** Details of chemical composition steel powder (analyzed by powder producer).

Elements	Fe	Cr	Ni	Mo	Mn	Si	Nb	C (Total)
Mass (%)	Balance	18.8	38.4	0.01	0.02	1.68	1.49	0.48

**Table 2:** Particle size results evaluated using laser diffraction analysis.

Samples	d_43_	d_m_	d_10_	d_90_	Span
	(m)				
FRESH	30.5	28.18	19.73	43.1	0.8
FRESH 950 °C	31.69	31.99	18.66	45.91	0.88
3×	30.18	27.95	18.74	43.67	0.88
3× 950 °C	29.16	31.68	14.75	44.31	1.04
6×	32.3	31.68	20.25	46.47	0.86
6× 950 °C	30.37	31.85	15.34	46.2	1.04

## Data Availability

The data presented in this study are available on request from the corresponding author.
